# Mother to child transmission of HIV in Brazil: Data from the "Birth in Brazil study", a national hospital-based study

**DOI:** 10.1371/journal.pone.0192985

**Published:** 2018-02-13

**Authors:** Rosa Maria Soares Madeira Domingues, Valeria Saraceni, Maria do Carmo Leal

**Affiliations:** 1 Laboratório de Pesquisa Clínica em DS/Aids, Instituto Nacional de Infectologia Evandro Chagas, Fundação Oswaldo Cruz, Rio de Janeiro, Brazil; 2 Coordenação de Análise da Situação em Saúde, Secretaria Municipal de Saúde do Rio de Janeiro, Rio de Janeiro, Brazil; 3 Departamento de Epidemiologia e Métodos Quantitativos em Saúde, Escola Nacional de Saúde Pública Sérgio Arouca, Fundação Oswaldo Cruz, Rio de Janeiro, Brazil; George Washington University, UNITED STATES

## Abstract

**Aims:**

to estimate the mother to child transmission (MTCT) of HIV among infected pregnant women identified in the "Birth in Brazil" study and to evaluate care practices provided in order to identify missed opportunities at preventing the MTCT of HIV infection in the country.

**Methods:**

Descriptive study using data obtained from the consultation of different databases: the “Birth in Brazil” study database and the Brazilian National Information Systems (NIS) databases. We used cases of pregnant women infected with HIV identified in the “Birth in Brazil” study, and cases of AIDS in children under 5 years old identified in the NIS, to estimate the MTCT of HIV infection in the country, with a 95% confidence interval. We also estimated the HIV cascade (HIV diagnosis; use of antiretroviral treatment (ART) during pregnancy, labour, and for the newborn; adequate care during childbirth considering viral load at birth; and no breastfeeding) using data from the same sources.

**Results:**

MTCT of HIV of 2.0% (95% CI 0.3%-13.8%). At birth, 84.0% of HIV infected woman showed a positive HIV diagnosis, 74.9% received combined ART during pregnancy, 80.7% received ART during childbirth, 77.1% received adequate care during childbirth, 86.8% of newborns received ART within the first 24 hours after birth, and 2.8% of newborns were breastfed. Considering all steps, 61.3% of the women (95% CI 48.3%-72.8%) received all available medical interventions. In the analysis restricted to women identified in the NIS, 65.3% (95% CI 48.0%-79.3%) of HIV infected women received all available medical interventions.

**Conclusion:**

Brazil has healthcare policies that guarantee free access to tests, ART and substitutes for maternal milk. However, missed opportunities to prevent MTCT of HIV were identified in at least one-third of women and may be making it difficult to reach HIV-elimination targets especially in the less developed country regions.

## Introduction

The mother to child transmission (MTCT) of HIV infection during pregnancy, delivery and breastfeeding is the leading cause of HIV infection in children. In the absence of preventive measures, MTCT of HIV can reach 25–40%, but the use of prophylactic measures, mainly combined antiretroviral drugs, can reduce MTCT to rates below 2% [1].

In 2001, member states at the United Nations General Assembly Special Session on HIV and Aids committed to reducing HIV infection in children by 20% by 2005 and 50% by 2010 and ensuring 80% of pregnant women in antenatal care received information, preventive services and treatment to reduce MTCT of HIV [2]. In 2010, member countries of the Pan American Health Organisation (PAHO) adopted goals for eliminating vertical transmission of syphilis and HIV by the year 2015, with HIV elimination impact goals expressed as the reduction in the incidence of HIV cases in children to ≤ 0.3 cases per 1000 live births and a vertical transmission rate ≤ 2%. In 2016, these goals were renewed and expanded upon by the "Action Plan for the Prevention and Control of HIV and Sexually Transmitted Infections (2016–2021)". Changes included the elimination of hepatitis B and Chagas' disease in the Americas [3].

In 2011, the “Global plan for the elimination of paediatric HIV infections” proposed the goal of eliminating paediatric HIV infections worldwide by 2015, and in 2014, the World Health Organisation (WHO) defined the criteria for validation of the elimination of vertical transmission of syphilis and HIV. For HIV infection, the indicators of impact are defined as the achievement of ≤ 50 new HIV infections in children per 100,000 live births, and a vertical transmission rate < 2% in cases without breastfeeding. As process indicators, the goals include achieving at least one prenatal visit for ≥ 95% of pregnant women, at least one HIV test for ≥95% of pregnant women during prenatal care, and the use of combined antiretroviral therapy by ≥90% of pregnant women with HIV [4]. In the Americas, the goal of combined antiretroviral therapy use by infected pregnant woman is ≥ 95% [3].

According to the latest WHO progress monitoring report [1], about 1.4 million (1.3–1.6 million) pregnant women from low and middle-income countries were still infected with HIV in 2013. In these countries, the HIV–MTCT rate fell from 26% (23%-29%) in 2009 to 17% (14%-19%) in 2013, with 160,000 fewer children with HIV, a reduction of 40% in the period 2009–2013. In Latin America, the HIV–MTCT rate decreased by 53% during the period 2010–2015, from 15% (11%-20%) to 8% (6%-10%). By 2015, 22 countries in the Americas region reported results that met the goals of eliminating HIV–MTCT [3].

In Brazil, it is estimated that approximately 0.38% of pregnant women are infected with HIV [5, 6], which corresponds to nearly 11,000 pregnant women being infected with HIV per year. Prenatal and childbirth coverage in the country is close to 100%, and tests, medication and maternal milk substitutes have been available for the prevention of MTCT of HIV since the 1990s. National studies have reported a reduction in MTCT rates in the country, with regional variations [7–10]. However, the MTCT rate is not routinely estimated by the Brazilian Ministry of Health (MS). Instead, the rate of AIDS detection in children less than 5 years old is used as a proxy indicator for monitoring HIV–MTCT. According to the latest epidemiological bulletin of the Brazilian Ministry of Health, there is a downward trend in the rate of detection of AIDS in the country among children less than 5 years old, with a 42.7% reduction in the rate in the period 2006–2015, albeit with regional inequalities [11].

The "Birth in Brazil" study is a national study with the objective of evaluating the quality of antenatal and delivery care in the country and the results of this care, with emphasis on the excessive number of caesareans, their determinants and perinatal outcomes. The study identified pregnant women with HIV from data recorded on the prenatal card and hospital charts, with the aim of better assessing the causes and consequences of unnecessary caesarean sections.

This study aims to estimate the MTCT of HIV among infected pregnant women identified in the "Birth in Brazil" study and to verify the care practices provided to identify missed opportunities for the prevention of MTCT of HIV in the country.

## Materials and methods

This is a descriptive study that uses data obtained from the consultation of different databases: the “Birth in Brazil” study database and the Brazilian National Information Systems (NIS) databases [“National System of Disease Notification” (SINAN), “National Network System of Laboratories of CD4 + / CD8 + Lymphocyte Count and Viral Load” (SISCEL) and “Logistic Control System of Medicines” (SICLOM)].

The “Birth in Brazil” study is a national hospital-based study conducted between February 2011 and October 2012. The study included 23,894 women hospitalised in 266 public and private hospitals located in 199 municipalities in all regions of the country. We considered all women who gave a live birth where the foetus was of any gestational age or weight, or a stillbirth where the foetus was of a gestational age greater than 22 weeks or weighing more than 500 grams, as eligible for the study. Further information about the study design and the sample calculation can be found in Leal [12] and Vasconcellos [13].

The National System of Disease Notification (SINAN) controls all diseases of compulsory notification in the country. It is stipulated that, in Brazil, all cases of "HIV in pregnancy", "Children exposed to HIV" and "AIDS in children" should be reported using a specific form for each disease. The "HIV in pregnancy" notification form contains information on the use of antiretroviral medication for women during pregnancy and delivery and for the newborn after birth. The Logistic Control System of Medicines (SICLOM) aims to manage the logistics of antiretroviral drugs (ARVs) in Brazil. All antiretroviral drugs provided to pregnant women with HIV, children exposed to HIV, and people (adults and children) living with HIV/AIDS are registered in this system. “The National Network System of Laboratories of CD4 + / CD8 + Lymphocyte Count and Viral Load” (SISCEL) has recorded the CD4 / CD8 counts and viral load among people living with HIV and AIDS since 1997 in the country. For this study, the Ministry of Health provided the nominal databases of SINAN, SISCEL and SICLOM after authors signed a term of responsibility form.

To identify cases of pregnant women with HIV in the "Birth in Brazil" study we collected data from prenatal cards and hospital records, according to the following criteria [5]:

1. HIV serological reagent results recorded on the prenatal card (two rapid tests or Elisa + immunofluorescence or Elisa + Western Blot);
  or
2. Records in medical records of any of the following situations:
  - Diagnosis of HIV infection;
  - HIV infection as an indicator for caesarean section;
  - Use of Zidovudine (ZDV) during labour and / or birth;
  - Use of ZDV syrup for the newborn;
  - Discontinuation of breastfeeding due to maternal HIV infection;
  - Registration of the diagnosis "child exposed to HIV".

To estimate the MTCT of HIV infection among pregnant women identified by the "Birth in Brazil" study, we researched AIDS cases in children less than 5 years old using SINAN during the period from 2011 to 2016. The Department of STD, AIDS and Viral Hepatitis of the Ministry of Health routinely cross-references the SINAN database with the Mortality Information System (SIM), the SISCEL system and the SICLOM system as sources for data collection. Thus, SINAN includes all cases of AIDS in children identified in the four existing systems containing information on HIV / AIDS infection.

To obtain information on the use of antiretroviral drugs by the pregnant women with HIV identified in the "Birth in Brazil” study, we searched for cases in SINAN “HIV in pregnancy” and the SICLOM. To obtain information about viral load close to delivery, we searched for pregnant women infected with HIV in the SISCEL. To identify the pregnant women infected with HIV at SINAN, SISCEL and SICLOM, we conducted searches for the period from 01/05/2010 to 31/10/2012. By including the 2010 period, we aimed to identify pregnant women who gave birth by February 2011 and who had been reported to SINAN, or registered at the SISCEL or the SICLOM during pregnancy in 2010. Initially, we planned to look for "children exposed to HIV" at SINAN. However, there was no database available for consultation regarding the “children exposed to HIV” cases.

The “Birth in Brazil” database was cross-referenced with SICLOM, SISCEL, SINAN “HIV in pregnancy” and SINAN “Aids in Children”. We applied the probabilistic record linkage method [14, 15] after sorting data. The matching variables for the first three datasets were: pregnant woman’s name, mother’s name, date of birth and city of residence. The matching variables for SINAN “Aids in Children” were: pregnant woman’s name as mother’s name, date of delivery and city of residence. We checked addresses of matching pairs to confirm the information. Due to the small number of matches, we manually searched for all children in the SICLOM/SISCEL databases.

Following the cross-reference, we constructed a final database including all cases of pregnant women infected with HIV identified in the "Birth in Brazil" study, all children diagnosed with AIDS identified in the NIS, and variables related to the protocols for the prevention of MTCT of HIV available at both sources. We obtained information regarding medication used during pregnancy from SICLOM and SINAN; information regarding the use of antiretroviral medication during labour and delivery and by the newborn after birth from SINAN and from hospital records; information on viral load tests results at the earliest date before childbirth from SISCEL and hospital records. We obtained all data relating to type of delivery (vaginal/forceps, antepartum caesarean section, intrapartum caesarean section), characteristics of the newborn (live/foetal loss/neonatal death, gestational age at birth, birth weight, Apgar at first and fifth minutes) and breastfeeding during hospital stay from the “Birth in Brazil" study database.

We calculated the MTCT of HIV infection, with a 95% confidence interval, as the proportion of AIDS cases in children under 5 identified in the NIS in relation to the total number of pregnant women diagnosed with HIV identified in the "Birth in Brazil" study. We also recorded the following indicators of care practices: a) the proportion of pregnant women infected with HIV who received at least one prenatal visit; b) the proportion of pregnant women infected with HIV who were diagnosed with the infection prior to the current gestation period or during prenatal care; c) the proportion of pregnant women infected with HIV who used antiretroviral medication during pregnancy and delivery; d) the proportion of children exposed to HIV who received antiretroviral medication in the first 24 hours of life; e) the proportion of newborns who were breastfed during hospital stay; f) the proportion of birth types considered adequate, that is, the type of birth (caesarean section or vaginal delivery) according to the viral load (<1000 copies ≥1000 copies, ignored) closest to childbirth.

The "Birth in Brazil" study used a complex sampling design that we took into account during all statistical analysis using weighting and calibration procedures for all the data (numbers and proportions) presented in this study. All analyses were performed using the software IBM SPSS Statistics for Windows, version 19.0 (IBM Corp., Armonk, NY, USA).

The “Birth in Brazil” study and the present study were approved by the Research Ethics Committee of ENSP/Fiocruz, report number 92/2010 and 1,647,494/2016, respectively. During the “Birth in Brazil” study, we obtained an informed consent statement before data collection. For the present study, an informed consent was not needed, as it was a database analysis. Only members of the study team accessed the nominal databases and we took all necessary care to ensure the confidentiality of the information.

## Results

The 74 pregnant women with HIV identified in the "Birth in Brazil" study had a mean age of 28 years, ranging from 17 to 42 years, with 73.7% in the 20–34 age group. Approximately 75% reported skin colour as ‘black’ or ‘mixed-race’, 50.1% had less than 9 years of schooling, 30.7% did not live with a partner, and 70.5% were not employed in paid work. More than one third of the women reported three or more previous pregnancies, 29.4% reported previous abortion and 34.3% had three or more previous births. Of the women with previous births, 11.6% indicated a previous preterm birth, 13.6% gave birth to a newborn with low birth weight, 4.9% showed cases of previous neonatal death and 5.0% previously gave birth to a stillbirth. Only 2.8% of pregnant women with HIV infection received childbirth care in private maternity services (Table 1).

**Table 1:** Social and demographic characteristics of women diagnosed with HIV infection during pregnancy. Brazil, 2011–2012.

Characteristics of women	n	%	CI 95%
**Maternal age**			
12 to 19 years	5	7.4	3.1–16.8
20 to 34 years	54	73.7	62.7–82.3
35 and over	15	18.9	11.7–29.0
**Skin color**			
White	18	25.1	17.8–34.2
Mixed	17	51.8	42.1–61.4
Black	38	23.0	15.1–33.5
**Schooling level (years)**			
0 to 8	37	50.1	37.1–63.0
9 to 11	20	27.0	15.5–42.8
12 to 15	15	19.8	11.5–32.0
16 or more	2	3.1	0.7–12.6
**Conjugal situation**			
Without partner	23	30.7	22.1–41.1
With partner	51	69.3	58.9–77.9
**Paid work**			
Yes	22	29.5	19.0–42.9
No	52	70.5	57.1–81.0
**Number of previous pregnancies**			
0	12	16.1	8.3–28.9
1	18	24.3	16.2–34.7
2	18	23.9	15.5–34.9
3 or more	26	35.7	24.2–49.1
**Number of previous births**			
0	15	20.5	12.3–32.1
1 or 2	33	45.3	32.6–58.6
3 or more	25	34.3	22.9–47.8
**Previous abortion** ^1^	22	29.4	19.8–41.3
**Previous preterm birth**^2^	9	11.6	4.9–24.8
**Previous low birthweight** ^2^	10	13.6	7.3–24.1
**Previous neonatal death**^2^	4	4.9	1.0–19.8
**Previous stillbirth**^2^	4	5.0	1.4–15.7
**Type of birthcare funding**			
Public	72	97.2	96.1–97.9
Private	2	2.8	2.1–3.9

CI = Confidence Interval.

^1^ In women with previous pregnancy,

^2^ In women with previous birth

Of the pregnant women with HIV, 95.8% received at least one prenatal visit, 54.3% started this care during the first gestational trimester, 75.1% had an adequate number of consultations (considering the gestational age at birth and a minimum of 6 consultations for a term, low-risk pregnancy), 84.0% were diagnosed with HIV before or during prenatal care and 74.9% received combined ART during pregnancy. Almost half of the pregnant women with HIV (45.2%) had no viral loads available at the end of pregnancy (17.8% not tested, 27.4% with results not informed), 11.3% had a viral load higher than 1,000 copies, and 43.6% had an undetectable viral load (Table 2). Among women using antiretroviral medication during pregnancy, these values were 30.4%, 15.0% and 54.5%, respectively.

**Table 2:** Prenatal care of women diagnosed with HIV infection during pregnancy. Brazil, 2011–2012.

Prenatal care indicators	n	%	CI 95%
**At least one prenatal care vist**	70	95.8	87.6–98.6
**Start of prenatal care**			
Up to week 12	38	54.3	41.1–67.0
Weeks 13 to 28	28	40.5	29.2–52.9
>Week 28	4	5.2	1.4–17.3
**Adequacy of number of consultations**			
Yes	51	75.1	61.3–85.2
No	17	24.9	14.8–38.7
**Diagnosis of HIV infection before or during antenatal care**	62	84.0	73.3–90.9
**ART during pregancy**			
Yes	55	74.9	62.9–84.0
No	12	15.9	8.4–28.2
Not informed	7	9,1	5.3–15.4
**Viral load near childbirth**			
< 1,000	32	43.6	30.3–57.9
> = 1,000	8	11.3	6.2–19.5
Not tested	13	17.8	10.0–29.7
Not informed	20	27.4	18.6–38.4
**Viral load near childbirth among women on ART**			
< 1,000	30	54.5	38.4–69.7
> = 1,000	8	15.0	9.2–23.5
Not tested	2	4,4	3.7–5.2
Not informed	14	26.0	14.7–41.9

CI = confidence interval; ART = antiretroviral treatment.

Of the total number of pregnant women with HIV, 80.7% used ZDV at birth and 86.8% of newborns received ART within the first 24 hours after birth. Approximately 13.4% of the pregnant women did not have information regarding ART administration during labour and 12.0% to the newborn. Restricting the analysis to the women identified in the SINAN “HIV in pregnancy”, the only system with information on ART use during childbirth and by the newborn, ART was applied during 90.2% of childbirths and 98.5% of newborns received this treatment (Table 3). Women not diagnosed with HIV prior to delivery did not receive ART during pregnancy and showed 63.2% application during delivery and 94.8% for the newborn. About one-quarter of the births were by vaginal delivery, 12.5% by intrapartum caesarean section and 61.3% by antepartum caesarean section. The analysis of the type of delivery according to the viral load close to the birth shows that, in total, 77.1% of HIV infected women received adequate care during delivery according to the viral load (Table 3). However, the rate of adequacy varied according to the viral load, with lower adequacy among women with an unknown viral load, who showed higher rates of vaginal birth (34.5%) and intrapartum caesarean section (13.7%). Among women with a viral load greater than 1,000 copies, 10.0% received intrapartum caesarean section and 90.0% antepartum caesarean section. The majority of women with an undetectable viral load received an antepartum caesarean section (63.8%), while 24.3% delivered vaginally and 11.9% via intrapartum caesarean section. One quarter of the infants born to pregnant women with HIV were preterm and 22.3% had low birth weight. Less than 3% of women reported breastfeeding during the hospital stay (Table 3).

**Table 3:** Characteristics of childbirth care and newborns of pregnant women diagnosed with HIV infection. Brazil, 2011–2012.

Childbirth and newborn characteristics	n	%	CI 95%
**Type of birth**			
Vaginal birth	19	26.2	17.4–37.4
Antepartum caesarean section	45	61.3	48.2–73.0
Intrapartum caesarean section	9	12.5	6.3–23.2
**Adequate type of birth according to viral load**	57	77,1	66,1–85,4
**ZDV during labour and childbirth**			
Yes	59	80.7	68.5–88.9
No	4	6.0	2.4–14.5
Not informed	10	13.4	6.9–24.4
**ZDV during labour and childbirth (SINAN only)**			
Yes	38	90.2	77.4–96.1
No	3	6.4	1.9–19.6
No informed	1	3.4	0.8–13.3
**ART administration to the newborn**			
Within the first 24 hours of life	64	86,8	76,7–93,0
After 24 hours of life	1	1.1	0.1–8.3
Not informed	9	12.0	6.1–22.2
**ART administration to the newborn (SINAN only)**			
Within the first 24 hours of life	41	96.5	87.0–99.1
After 24 hours of life	1	2.0	0.2–14.3
Not informed	1	1.5	0.2–11.3
**Gestational age**			
<37 weeks	19	25.6	17.5–35.9
≥ 37 weeks	55	74.4	64.1–82.5
**Birth weight**			
< 2.500g	17	22.3	13.8–34.1
≥ 2.500g	58	77.7	65.9–86.2
**Breastfeeding**	2	2.8	1.4–5.6

CI = Confidence interval, ZDV = zidovudine; ART = antiretroviral therapy; SINAN = National System of Disease Notification

**Fig 1:**
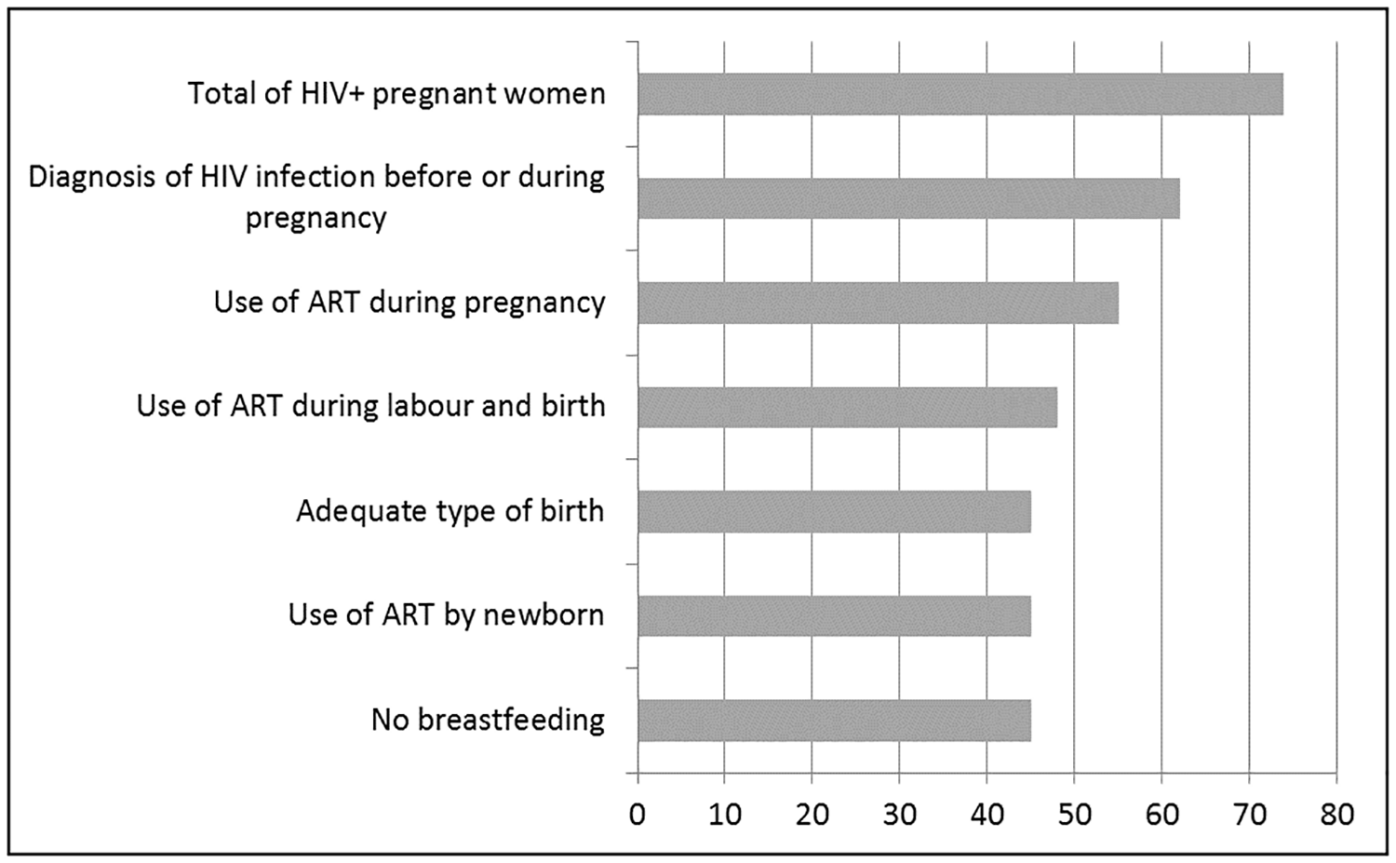
Cascade of interventions to prevent the mother to child transmission of HIV. Brazil, 2011–2012.

**Fig 2:**
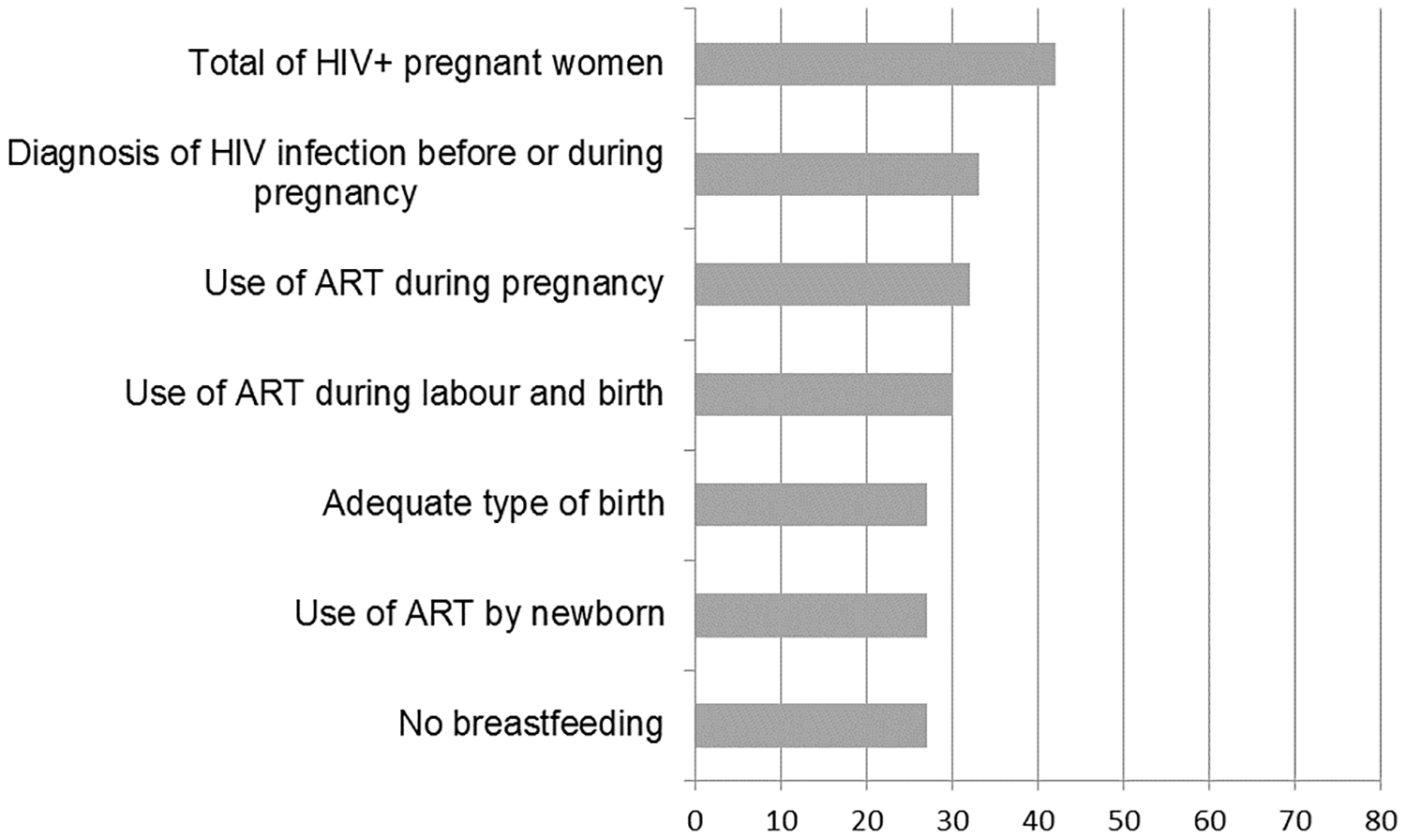
Cascade of interventions to prevent the mother to child transmission of HIV in women identified in the SINAN “HIV in pregnancy”. Brazil, 2011–2012.

Fig 1 shows the cascade of interventions to prevent the MTCT of HIV and our calculations of the proportion of each stage of intervention in relation to the total number of women who received the previous intervention. Fig 2 presents the same cascade of interventions but only for the pregnant women with HIV identified in the "Birth in Brazil" study and in the SINAN “HIV in pregnancy”. In both figures, the greatest missed opportunity is the diagnosis of HIV infection, a loss of 16% in Fig 1 and 20.7% in Fig 2. Considering all steps, including the type of delivery appropriate for the viral load level, 61.3% of the women (95% CI 48.3%-72.8%) in Fig 1 and 65,3% (95% CI 48,0%-79,3%) in Fig 2 received all available interventions.

We identified only one case of AIDS in children from the SINAN previously cross-referenced with the SIM, SISCEL and SICLOM systems, with no additional cases of children with HIV located in the searches we conducted in SISCEL and SICLOM. Having identified the case, we estimated a national MTCT of HIV of 2.0% (95% CI 0.3% -13.8%) and it was not possible to estimate the MTCT of HIV by region.

## Discussion

The "Birth in Brazil" study was conducted in maternity wards where more than 500 births take place each year. Since 99% of births in Brazil occur in hospitals, with more than 80% of them in institutions with more than 500 births/year, the results presented reflect the care practice provided to the largest contingent of Brazilian women. However, it cannot be extrapolated that the same applies for infected women who have had a miscarriage, who planned to give birth outside of a hospital or those who did so unintentionally, or who delivered in a smaller hospital.

In the "Birth in Brazil" study, pregnant women with HIV were identified from data recorded on the prenatal card or in hospital charts, and no blood collection was performed for serological tests or laboratory confirmation. Therefore, it is possible that errors occurred during the classification of HIV positive pregnant women, due to failures in these methods of data collection. However, the estimates of HIV infection obtained in the "Birth in Brazil" study are similar to those found in a nationwide study conducted in the same period (2010–2012), with blood collection for HIV serology [6].

We researched AIDS cases in children using the SINAN during the period 2011–2016. Some children included in the "Birth in Brazil" study born in 2012 would still not have reached 5 years old during this period. Therefore, it is possible that some cases could still be reported in 2017. The non-availability of the SINAN "Children exposed to HIV" database also limited the identification of cases. Because of these limitations, it is possible that we underestimated cases of AIDS in children under 5 years old.

In fact, the 2.0% MTCT of HIV estimated for the country was lower than the rate reported by the Brazilian government to PAHO in 2014, of 4.4% [3], and is within the limit of the elimination goal adopted by the Americas Region (vertical transmission rate ≤ 2%). The small number of cases resulted in an inaccurate estimate, varying from 0.3%-13.8%, and prevented the estimation of the MTCT by region in the country. However, these values are compatible with regional variation reported in local studies: rates of 2%-3% in studies conducted in the South [16, 17], Southeast [7, 8, 18–20] and Midwest [21, 22], and rates close to 10% in the North [10] and Northeast [9] regions of the country. The detection rate of cases of AIDS in children under 5 years old in the country follows this same pattern, with the Southeast, South and Midwest regions showing the biggest reduction trends; 73.2%. 63.4% and 82.5%, respectively, from 2006 to 2015. The northern region showed a smaller decrease, of 17.9%, from 4.1 in 2006 to 3.5 cases/100 thousand inhabitants in 2015, while the Northeast maintained the 2.4/100 thousand inhabitants in the same period [11].

Of the elimination goals related to the care process, only prenatal care coverage reached the desired value (≥95%). It is estimated that, in Brazil, 98.7% of pregnant women receive prenatal care [23]. In this study, 95.8% of pregnant women with HIV had at least one prenatal care appointment, a value higher than that observed by Miranda et al [24] in a study that evaluated women who were recorded in the SINAN “HIV in pregnancy”, where the coverage ranged from 79% to 91%.

The diagnosis of HIV is an essential step towards the prevention of HIV-MTCT, since it allows for the implementation of available clinical guidelines. HIV testing coverage in the country is estimated to be 80%, below the WHO target of coverage ≥ 95%, and is even lower in women with greater vulnerability to HIV [5]. In the present study, 84.0% of the pregnant women were diagnosed with HIV during pregnancy or before gestation. Opportunities to diagnose the infection were squandered in almost one fifth of cases. Among infected pregnant women, the application of combined ART during pregnancy was 74.9%, lower than the PAHO (≥ 95%) [3] and WHO (≥ 90%) [4] goals, and similar to the WHO reported value for low and middle-income countries (67% CI 95% 62% -73%) [1].

One of the challenges to prevent HIV-MTCT is achieving viral suppression in the short gestation period. In this study, even among women with ART during pregnancy, viral suppression was only achieved in approximately half of pregnant women, with 45.4% of women having a high (15.0%) or unknown (30.4%) viral load at birth. As SISCEL data on viral load are available to the public services only, it was not possible to obtain this data for 2.8% of the women cared for in private services. It is probable that for all other women assisted in public services with unknown viral load, there was not enough time to record the viral load before birth.

The early initiation of prenatal care is critical for the swift diagnosis of infection and timely start of antiretroviral therapy aiming the achievement of viral suppression. In addition, some studies indicate that early prenatal care initiation [25, 26] and the application of ART in pregnancy [27] are factors that will reduce the frequency of missed follow-ups of exposed women and children. This is possibly due to increased linking of women with health services and the care process. In this study, only 54% of pregnant women initiated prenatal care before the 12th gestational week, which hinders the early diagnosis of the infection and viral suppression.

Data presented on the use of antiretroviral medication during pregnancy, childbirth and administered to the newborn were obtained in the national information systems and in hospital records, and were subject to coverage limitations and variations in the accuracy of information. With the data available we estimated that only 61.3% of pregnant women received all interventions available, including ART during pregnancy and childbirth, and for the newborn; adequate care during childbirth according to the viral load; and suspension of breastfeeding. We obtained a similar result (65.3%) in the analysis restricted to women identified in SINAN “HIV in pregnancy”. In both scenarios, we found a similar care pattern in these services, we more than a third of pregnant women with missed opportunities to prevent HIV transmission during pregnancy and hospital stay for childbirth.

A study carried out with pregnant women documented in SINAN “HIV in pregnancy” [24] identified missed opportunities of HIV-MTCT prevention in all five regions of the country, but with higher frequency in the North and Northeast regions. Less missed opportunities were observed in women diagnosed with pre-gestational infection and higher in those who did not receive a diagnosis of HIV during pregnancy.

In this study, the absence of diagnosis of infection during pregnancy prevented the application of ART during pregnancy, reduced the possibility of ART during labour and childbirth, and led to inadequate care during childbirth: almost 50% of women with unknown viral load, and 10% of those with high viral load, gave birth vaginally or via intrapartum caesarean section. The antepartum caesarean section is the recommended technique of delivery for women with a viral load above 1,000 copies or with viral load ignored after the 34th gestational week. It should be noted that in women with an undetectable viral load, the caesarean rate was 75.7%. Although we have not evaluated the indicators for caesarean, it is unlikely that such a high rate resulted from clinical and/or obstetrical indications. Brazil has one of the highest caesarean rates in the world and it is estimated that a million unnecessary caesarean deliveries are performed per year [28]. It is possible that this high rate is due to an indiscriminate use of this procedure for pregnant women with HIV infection, regardless of the value of the viral load.

The primary goal of treating HIV is to achieve long-term viral suppression and improve the survival rates of people living with the infection. For this, it is necessary not only to identify and start antiretroviral treatment early, but also to link and retain people in health services so that they have the maximum benefit of antiretroviral treatment [1]. As such, in addition to the process indicators analysed in this study, other indicators mainly related to the retention of women and babies in services and adherence to antiretroviral therapy have been recommended [2].

Regarding children, in this study none of the children exposed to HIV were identified in SISCEL, a system that registers CD4 and viral load tests. Although the search was restricted to the period 2011–2012, the Brazilian Ministry of Health recommendation is that two viral load tests be performed in the first months of life to diagnose HIV. Therefore, the period we used would be sufficient for the identification of these exams. Likewise, no children were identified in the SICLOM, not even the one child diagnosed with AIDS. The absence of this information suggests flaws in the follow-up care routines of newborns exposed to HIV. Local studies have reported late diagnosis of HIV in children [29] and follow-up losses that can reach 30% in some places in the country [27].

This result follows a pattern observed worldwide. Complete information on the outcome of children exposed to HIV is not normally available, and the loss of follow-up information persists as a problem. Overall, early diagnosis of HIV has been achieved in less than 50% of the exposed children. This is the main reason for the low coverage of antiretroviral treatment in paediatric population. It is estimated that in 2013, less than a quarter of children aged 0–14 years living with HIV (23%) in low and middle-income countries received ART [1].

Brazilian clinical guidelines recommend the continuation of antiretroviral therapy after the termination of pregnancy for all women, regardless of the CD4 value and viral load. Although such data can be obtained from the nominal databases of SISCEL and SICLOM, monitoring indicators are not always available, making it difficult to assess care given to these women. It should be emphasised that women with HIV are more socially vulnerable [5], and thus specific strategies are required to link and retain this population in healthcare.

## Conclusion

Brazil has a prevalence of HIV in pregnant women of less than 1%, practically universal coverage of prenatal and birth care, and healthcare policies that guarantee universal and free access to tests, ART and substitutes for maternal milk. However, missed opportunities for preventing HIV-MTCT identified in at least one-third of women may be making it difficult to reach HIV elimination targets, especially in less developed regions of the country. Furthermore, failures in coverage and quality of data recording in national information systems make it difficult to monitor both measures that prevent HIV-MTCT and levels of retention of women and children in health services. Missed opportunities after hospital discharge following birth care were not evaluated in this study and should be investigated in the future.
